# Affect-inducing cues in risk messages: impact on collective negative emotions during public health emergencies

**DOI:** 10.3389/fpsyg.2026.1829692

**Published:** 2026-05-28

**Authors:** Tongtong Li

**Affiliations:** School of Journalism, Fudan University, Shanghai, China

**Keywords:** affect-inducing cues, collective emotions, negative emotion, public health emergency, risk message

## Abstract

**Introduction:**

This study examines how “affect-inducing cues” in risk messages—fear appeals, outrage factors, risk stories, and visual elements—affect collective emotions during public health emergencies.

**Methods:**

Using discussions about the COVID-19 variant “Xbb” on the Chinese Q&A platform Zhihu from December 2022 to January 2023, the study applies content analysis and fine-grained emotion analysis to measure these four cues, which serve as predictor variables. Additionally, a sentiment analysis method based on multiple sentiment lexicons and Chinese semantic rule sets is introduced to assess the prevalence of negative emotions among the message audience.

**Results:**

The findings reveal that the intensity of fear appeals in risk messages exhibits an inverted U-shaped relationship with the prevalence of negative emotions. Unexpectedly, risk stories reduce negative emotions, while the outrage factor and visual elements have no significant impact.

**Discussion:**

This study addresses the absence of a structural explanatory framework for collective emotional responses during public health emergencies, while practically providing new insights and empirical evidence for optimizing risk communication.

## Introduction

Infectious disease outbreaks trigger significant uncertainty, prompting the public to seek information (Jang and Baek, 2019) and engage in emotional sharing to vent anxiety (Rimé, 2009). These interactions often culminate in a “collective emotion” on social media, typically dominated by negativity as observed during the MERS, Ebola, and COVID-19 crises (Metzler et al., 2023; Song et al., 2017).

While these emotions reflect public narratives and can guide government risk communication, excessive negativity threatens social stability. Specifically, it can drive irrational behaviors like panic buying and herding (Gu et al., 2022; Liu et al., 2020), which disrupt emergency prevention and cause secondary disasters. Consequently, identifying the factors that exacerbate the spread of negative emotions is critical.

Currently, most research on collective emotions in public health emergencies focuses on documenting the evolution of emotional valence or discrete emotion types (e.g., Do et al., 2016; Metzler et al., 2023; Zheng et al., 2024). Although these studies have found that the prevalence of negative emotions can fluctuate with factors such as infection rates and government control measures, these conclusions are often event-specific, and a structural explanatory framework that consistently accounts for collective emotional responses remains absent.

This study addresses this gap by examining the properties of risk messages that the public is exposed to on social media. Grounded in the affect-inducing cues framework proposed by Visschers et al. (2012), this study incorporates four integral cues embedded in risk messages, namely fear appeals, outrage factors, risk stories, and visual images, as structural predictors of collective negative emotion on social media. While the Visschers framework has made important theoretical contributions to risk communication research, it was developed primarily in the context of one-way institutional communication and has not been directly tested in the interactive, user-generated content environments that characterize social media during public health emergencies. By subjecting the framework to empirical examination in this context, this study aims to advance the theoretical understanding of how risk message properties shape collective emotional dynamics in digital spaces, while also identifying the contextual boundary conditions under which the framework’s predictions hold.

Empirically, the study analyzes 228 discussion threads on Zhihu related to the COVID-19 XBB variant. Each discussion thread constitutes the unit of analysis. The initial post serves as the primary risk message, from which the four affect-inducing cues are measured. The comments generated in response are treated as expressions of collective emotional reactions, with the prevalence of negative emotions reflecting how these cues shape the collective emotional climate of the discussion.

### Social media and collective emotions

Collective emotions are defined by three core attributes: they are shared by a large group (Stephan and Stephan, 2000), emerge from interpersonal interactions (Goldenberg et al., 2020), and represent a collective reaction to a common stimulus (Von Scheve and Ismer, 2013). Crucially, collective emotions are not merely the sum of individual reactions; they are macro-level phenomena arising from dynamic processes where individuals perceive and mutually influence each other’s emotional states (Goldenberg et al., 2020). Thus, interaction is the fundamental prerequisite for collective emotion.

Social media facilitates this process by providing a real-time, highly interactive environment for immediate peer feedback (Skowron and Rank, 2014). As a central infrastructure of daily life, these platforms allow emotional posts and replies to trigger a cycle of mutual activation, sustaining the generation of collective emotions (Garcia and Rimé, 2019; Metzler et al., 2023; Thelwall and Kappas, 2014). Despite the dominance of text-based communication, digital text remains an effective medium for interpersonal emotional exchange (Garas et al., 2012). This study posits that analyzing social media conversation texts allows for the capture of natural emotional expressions and the tracking of the state of collective emotions.

### Empirical study of collective emotions in public health emergencies

During public health emergencies, public uncertainty regarding the event’s trajectory and dissatisfaction with institutional responses drive intensive emotional interaction on social media. Consequently, collective emotions have become a central focus of empirical inquiry. Although collective emotions possess three dimensions including quality, intensity, and time course (Chung et al., 2024; Goldenberg et al., 2020), current research primarily addresses emotional quality. Findings consistently show that negative emotions dominate social media during these crises. Specifically, the onset of outbreaks such as Ebola, MERS, and COVID-19 triggers a significant surge in anxiety, fear, and sadness compared to baseline levels (Cottingham, 2024; Li et al., 2020; Ofoghi et al., 2016).

Fewer studies have focused on the time course of collective emotions. It is widely accepted that collective emotions persist significantly longer than individual ones. Without social reinforcement, individual emotions typically return to baseline levels rapidly (Pellert et al., 2020). However, social media interactions facilitate a continuous mutual activation that creates emotional cascades, sustaining collective states over time (Goldenberg et al., 2020). Empirical evidence confirms that during prolonged crises like the COVID-19 pandemic, negative emotions on social media endure much longer than those triggered by isolated events such as natural disasters or terrorist attacks (Metzler et al., 2023).

While research on emotional quality has documented the prevalence and trajectory of negative emotions during crises, the message-level factors that systematically drive their emergence have received comparatively little attention. Some scholars have begun to move beyond description by examining specific message properties such as framing or narrative style (Huang et al., 2022; Klemm et al., 2019; Lee et al., 2019; Otieno et al., 2014). However, these efforts remain fragmented, often focusing on a single content characteristic in isolation and failing to account for the multidimensional nature of risk communication. Grounded in the affect-inducing cues framework proposed by Visschers et al. (2012), this study constructs an integrated explanatory framework for collective emotional dynamics during public health emergencies.

### Risk message properties

Visschers et al. (2012) distinguished between two types of affect-inducing cues in risk communication. Integral cues are emotional elements explicitly embedded within the risk message itself, directly tied to the risk content and capable of triggering immediate affective responses upon exposure. Incidental cues, by contrast, arise from the broader communication context rather than the message content, inducing emotions indirectly through situational factors such as processing fluency or graphic design. The present study focuses on integral affect-inducing cues, as these are directly controllable by risk communicators and more readily observable in naturalistic social media data. Visschers et al. (2012) identified four types of integral cues embedded in risk messages:

#### Fear appeals

Fear appeals, or threat appeals, are risk communication strategies that present adverse consequences to motivate audience compliance (Hovland et al., 1953; Witte, 1992). In this conceptualization, fear refers to the level of threat depicted within the message rather than the audience’s internal state (Tannenbaum et al., 2015). Consequently, high-fear appeals typically emphasize severity and susceptibility through vivid, intense language and somber visual tones (Lu and Yuan, 2023; Skurka et al., 2018).

Yet despite this message-level conceptualization, the audience’s internal affective response remains an essential component of fear appeal theory. Grounded in the Extended Parallel Process Model (EPPM) (Witte, 1992), negative emotions serve as pivotal variables between exposure to fear appeal messages and subsequent risk-coping responses. While Witte initially focused on fear, subsequent theoretical expansions have incorporated anxiety as a distinct emotional variable within the model (So, 2013). Empirical evidence consistently demonstrates that high-threat messages elicit greater fear and anxiety than low-threat ones (Witte, 1994; So et al., 2016). Beyond fear and anxiety, however, such messages can evoke a broader spectrum of negative states including sadness and anger (Dillard et al., 1996), making their collective emotional impact a critical area for investigation.

#### Outrage factors

Sandman (1993) defines risk as the sum of “hazard” and “outrage.” While hazard represents a technical assessment of severity and probability, outrage reflects the public’s emotional perception of risk through factors often overlooked by experts. Conceptually, outrage involves a non-technical evaluation that triggers significant emotional responses (Ju et al., 2015). Common outrage characteristics include immoral behaviors, institutional misconduct, and the portrayal of victims (Grobe et al., 1999; Lachlan and Spence, 2010). Covello and Sandman (2001) identified 20 specific outrage factors, such as voluntariness, controllability, fairness, and catastrophic potential. Risk messages prominently featuring these factors are likely to elicit intense collective emotions, including anger, fear, and distrust (Sandman et al., 1993; Wiedemann et al., 2003).

#### Risk stories

Risk stories depict specific scenarios that enable audiences to construct mental models of risk, incorporating concrete characters, motives, and the causal sequences leading to adverse outcomes (Palmlund, 1992; Visschers et al., 2012). The inherent concreteness of these narratives enhances risk availability and the perception that the threat is beyond personal control (Hendrickx et al., 1989). As an affect-inducing cue, risk stories are distinct in their requirement to portray the emotional experiences of victims. By conveying a specific emotional tone, these narratives can trigger various negative collective states, including fear, indignation, and compassion (Wiedemann et al., 2003; Visschers et al., 2012).

#### Visual elements

Vision serves as a primary sensory channel in risk communication (Xie et al., 2011). Based on the “Risk as Feelings” hypothesis, emotional responses to danger depend heavily on the vividness of the imagined consequences (Loewenstein et al., 2001). Generating such vivid mental imagery elicits stronger negative emotions than verbal processing alone (Holmes and Mathews, 2005). Visual elements, especially photographs, are highly effective at evoking this imagery by depicting threatening consequences or severe scenarios that simulate real-world experiences (Cameron and Chan, 2008; Qin et al., 2022). Consequently, the more vivid the imagery and the more tense the portrayed scenario, the more intense the resulting negative emotions (Damasio, 1994; MacInnis and Price, 1987).

This study attempts to explore the impact of these four types of affect-inducing cues on the quality of collective emotions:

*H1*: The stronger the fear appeal in risk messages, the more prevalent the induced negative emotions;

*H2*: The more the outrage factors in risk messages, the more prevalent the induced negative emotions;

*H3*: Risk messages containing risk stories induces more prevalent negative emotions compared to risk messages without risk stories;

*H4*: The more photos included in risk messages, the more prevalent the induced negative emotions.

## Methods

### Zhihu’s platform background

Data for this study were collected from Zhihu, one of China’s largest knowledge-sharing platforms, operating on a question-and-answer format comparable to Quora. Unlike microblogging platforms such as Weibo or X, where brevity and rapid information diffusion are characteristic, posts on Zhihu are expected to be substantive and well-reasoned, with users providing detailed analysis and explanations rather than brief assertions (Ru and Hu, 2016). While visual content is present on the platform, it plays a far less central role than on video-driven platforms such as YouTube, with text-based responses remaining the dominant mode of communication. Its user base skews toward highly educated, middle-class individuals in China, with approximately 80% holding a bachelor’s degree or above (Zhang, 2020), and it has established itself as a significant venue for civic engagement and deliberation on socio-political issues in China (Peng et al., 2022). These characteristics make Zhihu particularly suitable for examining public opinion and emotional dynamics within a Chinese context.

Since the onset of the COVID-19 pandemic, Zhihu has hosted extensive public discussions on a range of pandemic-related topics, including domestic and international containment policies, vaccination debates, and the contested efficacy of traditional Chinese medicine (e.g., Luo et al., 2021; Peng and Chen, 2021; Su and Li, 2026). Scholarly analyses of these discussions have demonstrated Zhihu’s value as a source of naturalistic data for studying public opinion and emotional responses during health crises (e.g., Li et al., 2022; Peng et al., 2020). The present study builds on this established research tradition by examining how risk messages about the XBB variant shaped collective emotional responses on the platform.

### Social context and data collection

In late 2022, China introduced 10 new measures to optimize its COVID-19 response, including reducing mass nucleic acid testing and allowing home quarantine for asymptomatic and mild cases (Xinhua News, 2022). Subsequently, in January 2023, China updated its COVID-19 diagnosis and treatment protocol, no longer identifying suspected cases or requiring positive cases to quarantine at designated venues (Xinhua News, 2023). During these 2 months, COVID-19 infections in China peaked (Global Times, 2023). Against this backdrop, the emergence of the XBB variant triggered widespread concern on social media, fueled by rumors linking the strain to gastrointestinal symptoms.

On Zhihu, the platform features a dedicated “Topics” section that aggregates discussion threads related to a specific subject. Each thread is centered on a question or a discussion prompt that serves as its title. This title is followed by an initial post providing a substantive response, under which other users can contribute comments. During the period between late 2022 and early 2023, discussions under the topic “Mutant strain XBB is coming” surged, accumulating over 63.9 million views. This study collected data from this topic via a Python-based web crawler operating through a registered personal account, which accessed publicly visible discussion threads and associated comments. No private user information was collected, and all data were anonymized prior to analysis to protect user privacy. The final sample comprised 228 valid threads and 12,894 associated comments from December 20, 2022 to January 27, 2023.

### Measuring comment-level negative emotion prevalence

The collective emotional state of each thread is assessed through the prevalence of negative emotions in the comment section. This serves as the outcome variable, operationalized as the proportion of negative comments among comments. To identify the emotional valence of individual comments, a sentiment analysis method grounded in multiple sentiment lexicons and Chinese semantic rule sets is employed (Wang et al., 2015; Wu and Lu, 2019). While originally developed for microblogging platforms such as Weibo, the approach is highly applicable to this dataset because 98.3% of the collected Zhihu comments are under 150 characters. This alignment in text length ensures the method is well-suited for capturing the emotional nuances of the sampled Zhihu discussions.

The analytical process begins with lexicon expansion: using word frequency, inside coupling, and neighboring character entropy as criteria, new affective words were extracted from the comment corpus and assigned sentiment polarities via point-wise mutual information, yielding a domain-specific lexicon tailored to pandemic discourse. Semantic rules were then applied at the word, clause, and sentence levels to compute an overall polarity score for each comment, with a positive score indicating positive sentiment and a negative score indicating negative sentiment, yielding a three-way classification of positive, negative, or neutral. Comprehensive details are available in the Supplementary materials S1.

### Measurement of predictor variables

The following four affect-inducing cues were each measured from the initial post of each thread.

#### Fear appeals

Fear appeals are quantified by the intensity of the “fear” emotion in the text (Widmann, 2022). Following Qiao et al.’s (2021) fine-grained emotion analysis, the intensity of fear in a thread’s initial post is calculated as:

$$E_{\text{fear}}=\sum_{i=1}^{k}{\left(-1\right)}^{\mathrm{Oi}}a_{i}p_{i}m$$ (1)

In Equation 1, K is the number of “fear” words; i represents each such word; O_i_ denotes negation relations; a_i_ is the degree adverb weight and p_i_ represents emotion intensity. The weight m adjusts for negation-degree adverb interactions, reducing intensity by 50% when negation precedes a degree adverb (e.g., “not very happy”), otherwise remaining 1 (Qiao et al., 2021).

#### Outrage factors

This study employed content analysis to measure outrage factors in the risk messages. A coding scheme was developed based on Covello and Sandman (2001) and Li and Zhong (2022), which was then refined to ensure conceptual distinctiveness and contextual relevance for user discussions on the COVID-19 Xbb variant in China in 2023. The final coding scheme included 12 factors (see Table 1). Each factor was coded as present (“1”) or absent (“0”), and the total outrage factor score was calculated by summing these values. The detailed factor selection and exclusion criteria are provided in the Supplementary materials S2.

**Table 1:** Content analysis coding table for outrage factors (Yes = 1; No = 0).

**Outrage factors**	**Statement**
Voluntariness	The “Xbb” strain or COVID-19 virus is an involuntary, imposed, and unavoidable risk.
Controllability	Whether or not we get infected is beyond our control.
Familiarity	The risks posed by the “Xbb” strain are unfamiliar to us.
Fairness	People with lower socioeconomic status are at greater risk during the COVID-19 pandemic.
Catastrophic potential	The “Xbb” strain could cause large-scale harm to people.
Delayed effects	The harm caused by the “Xbb” strain to the human body only appears after some time.
Effects on children	The negative impact of the “Xbb” strain on children is much greater than on adults.
Victim identity	The “Xbb” strain poses greater harm to specific groups of people compared to the general population.
Dread	The “Xbb” strain makes people feel fearful.
Accident history	Risks like the COVID-19 virus have threatened humanity in the past.
Reversibility	The damage caused by the “Xbb” strain to individuals and society is irreversible.
Personal stake	The “Xbb” strain or the COVID-19 pandemic has a greater negative impact on me and my family than on others.

#### Risk stories

The presence of risk stories in the initial post of a thread is also determined using content analysis. If the main post includes descriptions of the infection experience and emotional reactions of a COVID-19 patient or the prevention experience and emotional reactions of a close contact of a case, the post is coded as 1; otherwise, it is coded as 0.

#### Visual elements

The measurement of visual elements focused on counting the number of photographs in the initial post. As an affect-inducing cue, visual elements are characterized by their ability to depict tense risk situations or severe consequences. Accordingly, the photographs included in this study were those that provided direct visual depictions of infection symptoms, patient treatment processes, hospital response scenes, and daily life under pandemic control measures. In contrast, visual content conveying only objective or neutral information—such as statistical charts, research illustrations, or screenshots of expert tweets—was excluded from the count and not treated as an affect-inducing visual cue.

To ensure reliability in coding “outrage factors,” “risk stories,” and “visual elements,” this study conducted an inter-coder reliability test. A random sample of 100 initial posts was selected. Subsequently, two doctoral students in journalism and communication were employed to conduct the content analysis. Reliability testing showed that the coefficients for all variables exceeded 0.60 (Table 2), reflecting a dependable level of intercoder agreement (Landis and Koch, 1977), and formal coding could proceed.

**Table 2:** Intercoder reliability statistics.

**Variables**	**Cohen’s kappa**
Outrage factors	
*Voluntariness*	0.80
*Controllability*	0.83
*Familiarity*	0.69
*Fairness*	0.76
*Catastrophic potential*	0.70
*Delayed effects*	0.77
*Effects on children*	0.78
*Victim identity*	0.75
*Dread*	0.79
*Accident history*	0.67
*Reversibility*	0.80
*Personal stake*	0.73
Risk Stories	0.90

**Variables**	**Krippendorff’s alpha**
Visual elements	0.75

### Control variables

Due to the dynamic nature of collective emotions, their prevalence can fluctuate as discussions evolve (Goldenberg et al., 2020; Parkinson, 2011). To isolate the impact of message cues from the effects of ongoing interaction, this study controls for discussion duration (number of days).

Finally, multiple regression analysis is employed using the four affect-inducing cues as predictors and the prevalence of negative emotions as the outcome variable.

## Results

### Descriptive analysis and illustrative examples

**Figure 1:**
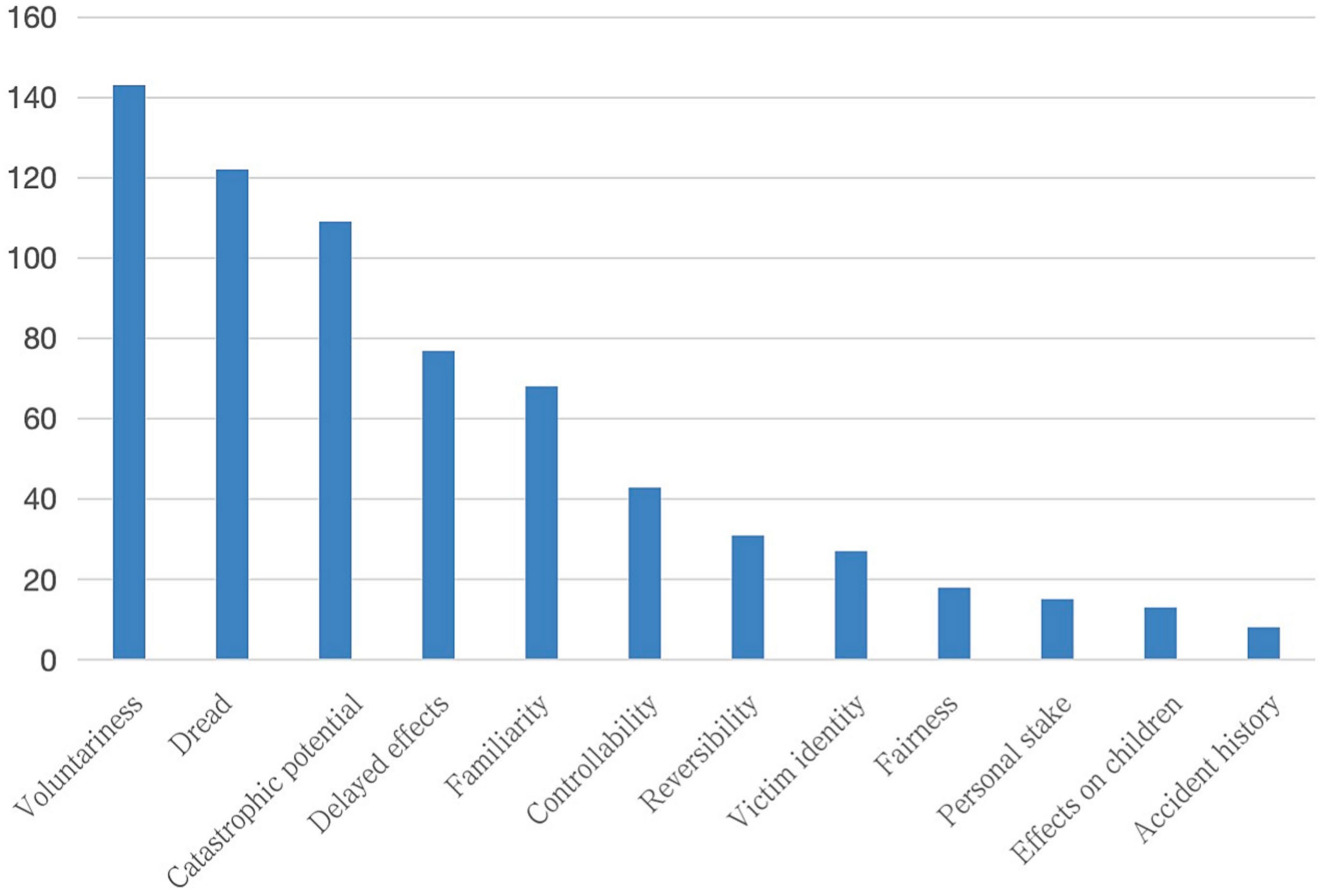
Frequency of each outrage factor.

Sentiment analysis of 12,894 comments identified 3,949 positive, 4,162 negative, and 4,783 neutral responses. The descriptive statistics for the predictor and outcome variables are summarized in Table 3. The distribution of outrage factors (Figure 1) shows that “voluntariness,” “dread,” and “catastrophic potential” were the most frequent cues, reflecting concerns over involuntary risk and widespread impact. Conversely, “fairness,” “personal stake,” “effects on children,” and “accident history” appeared least frequently. Descriptive results for visual elements show that risk information on the Zhihu platform did not widely utilize imagery for the visual concretization of threats, as evidenced by a mean score of 0.26 for these elements.

**Table 3:** Descriptive statistics of variables.

Variable	Mean	Standard deviation
Fear appeals	27.8	54.1
Outrage factors	2.96	1.73
Visual elements	0.26	1.28
Duration	8.32	8.43
Negative emotions proportions	31.91%	16.11%

	***N* (Coded as 1)**	***N* (Coded as 0)**
Risk stories	46	182

Fear appeals varied considerably in intensity across the sampled threads. Threads were categorized into low, moderate, and high groups based on tertiles of the fear appeal scores (see Table 4 for illustrative examples). Low fear appeal posts typically presented technical data or research findings in a measured tone, expressing little concern about the XBB outbreak. Moderate fear appeal posts acknowledged the potential harm of the variant and issued general health warnings, without dwelling on vivid or distressing details. High fear appeal posts emphasized the variant’s formidable immune evasion and mutation capabilities, often invoking a sense of helplessness against an uncontrollable threat.

**Table 4:** Representative examples of initial posts at low, moderate, and high fear appeal intensity.

**Fear appeal intensity**	**Original post (Chinese)**	**English translation**
Low (score = 0)	我觉得大家现在不用过于担心XBB，对于大多数人来说，下一次感染可能会在清明节前后，到时候是什么新变种谁又说的清楚。不过多备点感冒药、咳嗽药终归没有坏处。	I do not think everyone needs to worry too much about XBB right now. For most, the next infection might occur around the Qingming Festival. Nonetheless, it does not hurt to stock up on some cold and cough medicine.
Low (score = 2)	……XBB.1.5在美帝的异常增速也是咱们这边儿首先发现的:(分析图表)另外某位高赞朋友表示”还没有关于XBB.1.5的学术论文”……论文其实是有的啦，暂时还没发表而已，知乎这边咱十几天前就剧透过:(分析图表)更早的剧透则出现在其他地方，还顺带做了了一丢丢分析:(分析图表)现在关于XBB.1.5的已知情况，大致就是上面这些了吧。	... The unusual growth rate of XBB.1.5 in the US was first detected on our end: (analytical charts) A highly upvoted user claimed there are no academic papers on XBB.1.5 yet...there actually are, they just have not been published yet. We previewed this on Zhihu ten-plus days ago: (analytical charts) Even earlier previews appeared elsewhere, with some brief analysis: (analytical charts) What we currently know about XBB.1.5 is roughly summarized above.
Moderate (score = 15)	不管什么病毒，传播路径就那么几种。国家防疫政策个人无法左右，但尽全力做好个人和家庭成员的防护还是做得到的，不要跟傻子一样躺平，尽人事后听天命，仅此而已。傻子感染10轮，你只感染3轮，那就是赢了。BTW, 家庭和国家一样，做到统一认识是不容易的，防线从最薄弱处被突破，老年人很难搞定，可以收集多方面的负面消息来吓唬他们……	No matter the virus, there are only a few transmission paths. Individuals cannot control national prevention policies, but it’s possible to do one’s best to protect oneself and family members. Do not just “lie flat” like a fool; do your best and leave the rest to fate, that’s all. If a fool gets infected 10 times and you only 3, you have won. BTW, it’s not easy for a family to reach a consensus, just like a country. Defenses are breached at the weakest point; the elderly are hard to manage, so you can collect various negative news to scare them...
Moderate (score = 18)	感染率10-30%，病死率0.09%--0.16%，那么，中国总人口数*(10%OR30%)*(0.09%OR0.16%)，最小值是127080，最大值是677760。意味着12万——67万人感染新冠死亡。这还是没考虑医疗条件，医疗挤兑的保守估算……那么: 做好防护，自求多福	With an infection rate of 10–30% and a case fatality rate of 0.09–0.16%, multiplying China’s total population by these figures gives a minimum of 127,080 and a maximum of 677,760 deaths. That means 120,000 to 677,000 people could die from COVID-19. And this is a conservative estimate that does not account for healthcare capacity or medical system overload... So: protect yourself and hope for the best.
High (score = 31)	.所以你不要以为，感染过一次就再也没事儿了，无所畏惧了。因为一是抗体消失，二是免疫逃逸，你不做好防护，后续的感染会持续不断。直到病毒彻底把宿主消灭.所以就算是感染过的人，你也不要觉得高枕无忧。现在事实进一步证明了，我们中的大多数人会反复感染，直到生命终结。如果能够避免感染高峰和重症高峰，我们的成活率会高些。否则结果不会太好.	... So, do not assume that you are ‘done’ or invincible just because you have been infected once. Due to waning antibodies and immune evasion, infections will continue to recur if you do not take precautions, persisting until the virus completely eradicates its host. Even those who have recovered should not succumb to a false sense of security. The facts have further proven that most of us will face repeated infections throughout our lives. Our survival rate will only improve if we can avoid peaks of mass infection and severe cases; otherwise, the outlook is grim...
High (score = 106)	...原本国内这个巨大的培养基被排除在修罗场之外，诸多病毒变异株被拦在国门外卷生卷死，无从染指国内，只漏进来一些掉队的弱鸡(指BA.5.2、BF.7;8月成都的BA.2.76之类已经退场)……然而，国内这块处女地终究融入了全世界的大修罗场-----对我们人类来说，BA.5.2、BF.7与XBB.1、BQ.1.1这些绝顶高手之间没有交叉免疫，感染了前两种病毒变异株后，还是会感染后两种毒株的——当然也还会感染其他新变异株，运气不好一年能来个三四次。面对纷至沓来层出不穷的病毒变异株，我们该怎么办呢?...	... Originally, this massive domestic “culture medium” was excluded from the “Asura Field” (battlefield). Numerous variants were blocked at the border, competing fiercely with each other without reaching us, only letting in some “weaklings” (referring to BA.5.2, BF.7, etc.). However, this “virgin land” has finally merged into the global Asura Field. For us humans, there is no cross-immunity between “top masters” like BA.5.2/BF.7 and XBB.1/BQ.1.1. After being infected by the former, you will still be infected by the latter—and of course, other new variants. With bad luck, it could happen three or four times a year. Faced with an endless stream of emerging variants, what can we do?...

Risk stories, which appeared in approximately 20 percent of the threads (Table 3), served to ground the abstract health threat within the context of individual life paths. Representative examples of these narratives included detailed accounts of illness progression:

Xbb should not be underestimated. I lived in the United States for 3 years without catching the virus, relying on three Pfizer doses to avoid the original strain, Delta, and Omicron. However, I could not escape Xbb. Let me share my Xbb experience and perspective. Symptoms appeared last Sunday, which was the first day. Symptoms included a slight cough and dry throat. I did not think it was COVID so I showered as usual. Later the illness worsened. I tried to sleep after taking a packet of Ganmaoling but failed. While brushing my teeth, the dry throat and pharyngitis caused vomiting. I also experienced nasal congestion and sore throat.

Other narratives depicted the struggle to access medical resources during the peak of the infection:

If anyone tells me again that this is just a minor cold, I will feel like killing someone! I have used Ibuprofen, Acetaminophen, fever patches, and ice cubes. The queues at community health centers and small clinics are hundreds of meters long, and I simply cannot stand. I have no choice but to come back and endure it. Both medicinal and physical cooling methods have been used, and at this point, the fever patch loses its effectiveness within ten minutes.

### Multiple linear regression

**Figure 2:**
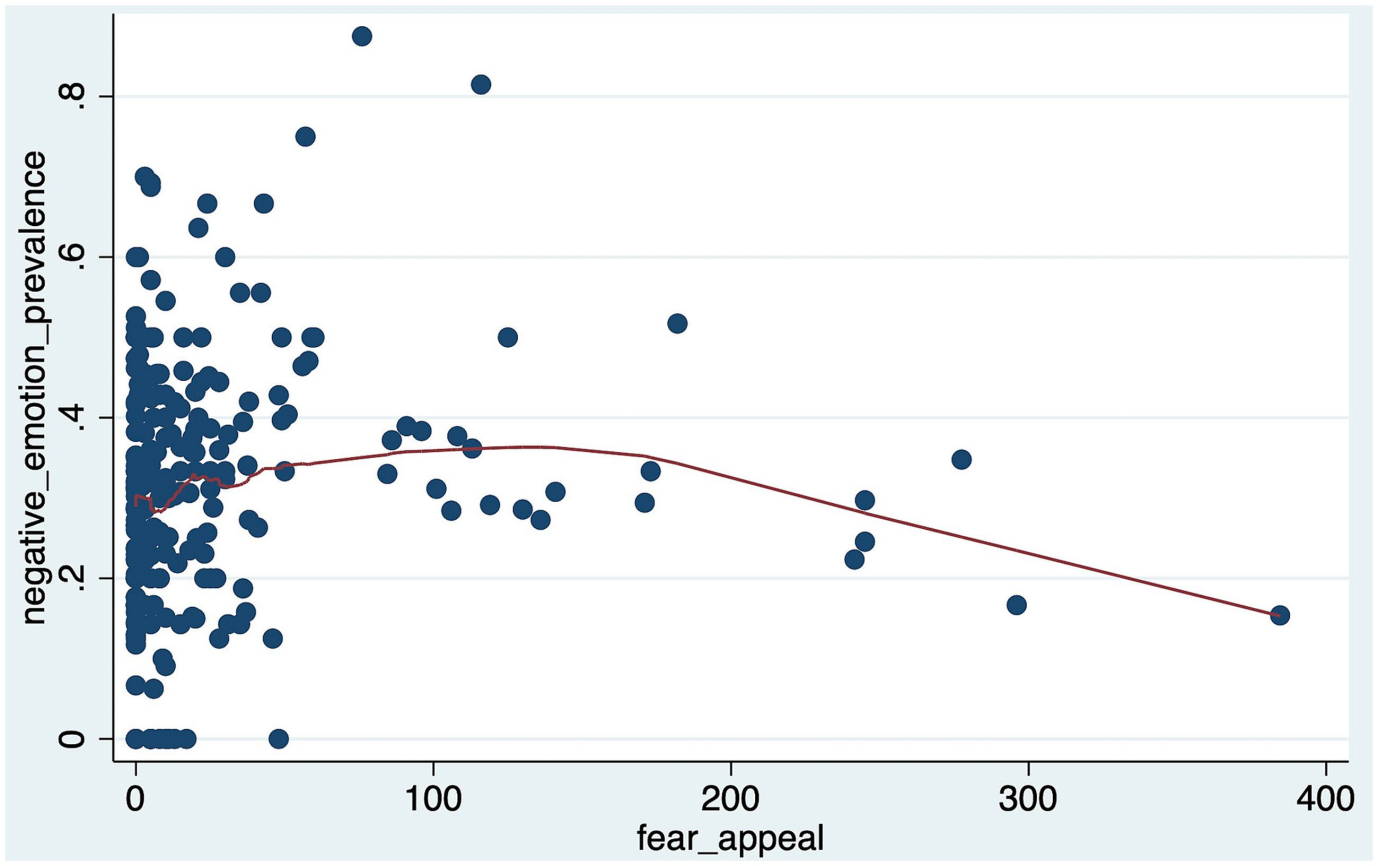
Fear appeal and negative emotion prevalence: A lowess smoothed fit.

The assumptions of linear regression require that the outcome variable and each predictor variable have a linear relationship. When tested using Stata software, this study found that fear appeals were not linearly related to the prevalence of negative emotions but instead showed a U-shaped relationship (Figure 2). Therefore, the quadratic term of the “fear appeals” variable was included in the model for correction.

Subsequently, to rule out the impact of multicollinearity, the study calculated the variance inflation factors (VIF) for all variables. Diagnostic results indicated that the VIF values for fear appeals and their quadratic term exceeded 5 (6.80 and 6.48, respectively), suggesting a clear multicollinearity problem. This collinearity primarily arose from the mathematical association between a variable and its squared term. To address this issue, the fear appeal variable was mean-centered by subtracting its mean from each original value before computing the quadratic term (Cohen et al., 2013). After centering, the VIF values for fear appeals and their quadratic term decreased to 4.69 and 4.28, respectively.

The model results (Table 5) indicate that affect-inducing cues do influence the prevalence of negative emotions. Regression analysis shows that the standardized coefficient for the mean-centered fear appeal variable is 0.15 (*p* < 0.001), suggesting that, after controlling for other variables, an increase in fear appeals is significantly and positively associated with a higher prevalence of negative emotions in the comment section. However, the coefficient for the squared term of fear appeals is negative (*β* = −0.44, *p* < 0.01), indicating that the relationship between fear appeals and the prevalence of negative emotions is not purely linear but follows an inverted U-shaped pattern. Specifically, as the level of fear appeals in risk messages increases, their effect on promoting negative emotions initially strengthens. Yet, beyond a certain threshold, further intensification of fear appeals may slow the growth of negative emotions or even lead to a decline. Based on these results, H1 is partially supported.

**Table 5:** Linear regression analyses for affect-inducing cues predicting negative emotion prevalence (*n* = 228).

Variable	Negative emotions proportions				
	*B*	SE	95% CI	*β*	*p*-value
Control variable					
Duration	−0.26	0.12	[−0.50, −0.01]	−0.14	<0.05
Predictor variable					
Fear appeal	0.15	0.04	[0.07, 0.23]	0.50	<0.001
Fear appeal*Fear appeal	−0.0006	0.0002	[−0.0010,-0.0003]	−0.44	<0.01
Outrage factor	0.07	0.63	[−1.18, 1.31]	0.007	0.92
Risk stories	−5.72	2.60	[−10.84, −0.60]	−0.15	<0.05
Visual elements	−1.26	0.93	[−3.10, 0.58]	−0.10	0.18
*R* ^2^	10.22%				
*F* (6, 221)	4.19, *p* < 0.001				

In addition, risk stories had a significant effect on the outcome variable, but the direction of the effect was opposite to H3. Risk messages containing risk stories (1 = 20.18%, 0 = 79.82%) elicited, on average, 5.72% fewer negative emotions than those without risk stories (*β* = −0.15, *p* < 0.05). Visual elements (*β* = −0.10, *p* > 0.05) and outrage factors (*β* = 0.007, *p* > 0.05) were not significant predictors of negative emotions. Therefore, H2 and H4 were not supported.

## Discussion

This study explores the impact of four affect-inducing cues in risk messages on audience emotional responses. Three main findings emerge: fear appeals exhibited an inverted U-shaped relationship with negative emotions; risk stories unexpectedly attenuated rather than amplified negative emotions; and outrage factors and visual elements showed no significant effects. These findings present a nuanced picture of how risk message properties shape collective emotions, with some cues operating as expected, others producing surprising reversals, and still others failing to manifest the effects theorized in prior research.

### Fear appeals: an inverted U-shaped effect explained by fear control

The inverted U-shaped pattern partially aligns with prior studies which typically use experimental methods to examine how varying fear appeal levels, such as low, medium, and high, influence emotions (Yoon and Tinkham, 2013; So et al., 2016). These studies found that higher fear appeals induce more fear and anxiety. Similarly, this study confirms a positive correlation between fear appeals and negative emotions up to a certain threshold, beyond which fear appeals have a counterproductive effect.

In previous experimental studies, the level of fear appeal was not treated as a continuous variable. Due to ethical constraints, it is unclear whether the levels used in those studies were sufficiently strong or merely ranged from low to moderate (Dillard and Li, 2020), a factor which potentially hindered the observation of effects within the high level range. This methodological limitation might account for the absence of the inverted U shaped correlation in prior research.

Beyond this methodological explanation, the emergence of this inverted U shaped relationship can be further elucidated by the mechanisms of the EPPM regarding how emotions shape coping strategies, which subsequently modulate the emotional state itself. While initial exposure to fear appeals triggers negative affect, the interplay between cognition and emotion dictates the coping process, which in turn feeds back into the emotional experience. The model suggests that fear appeals trigger two appraisals, specifically threat appraisal and coping appraisal. When the perceived threat becomes overwhelming but efficacy remains low, individuals experience excessive fear. This experience prompts a shift from danger control to fear control, which is a process focused on managing internal distress rather than the external risk. This process encompasses defensive reactions such as message denial or avoidance (Witte, 1992, 1994, 1996). Consequently, while fear is typically conceptualized as the driver of the fear control process, it can also be viewed as its consequence. This perspective aligns with the pivotal finding by Witte (1994) that fear levels and fear control processes are negatively correlated. Although one might initially expect a positive correlation because stronger fear should theoretically trigger more intense control processes, Witte (1994) argued that this negative relationship reflects a core tenet of the EPPM, namely that the activation of fear control effectively reduces the felt fear.

This underscores the critical importance of temporal dynamics in emotional measurement. As Li et al. (2024) emphasize, fear is a dynamic rather than static process characterized by a rise and fall pattern. Individuals’ fear typically peaks after processing a threat and then decreases following exposure to coping mechanisms, or in the case of fear control, after the activation of defensive maneuvers. Because our measurement via Zhihu comments likely captures the post control emotional state, the observed decline at high threat levels may represent the empirical trace of successful fear control. Consistent with the argument for dynamic data (Dillard et al., 2017; Li et al., 2024), our results suggest that static measures may miss the peak arousal phase and instead record the outcome of defensive blunting.

Beyond traditional defensive strategies such as message denial or psychological reactance which often require self reported data for identification (Popova, 2012), humor has been identified as another key fear control strategy that can be manifested directly in social media discourse (Abril et al., 2017). Humor represents a form of mockery that allows users to engage with a threat while simultaneously defusing its emotional impact (Abril et al., 2017; Steele, 2012). In the present study, a closer reading of comments within threads located in the declining phase of the inverted U-shaped distribution, where fear appeal intensity exceeded the threshold beyond which negative emotion prevalence begins to decrease, revealed humorous responses with these characteristics. Representative examples include self-deprecating exaggeration of protective measures: “As someone who does not rely on looks for a living and has a high alcohol tolerance, I always play it safe by spraying alcohol directly on my face [cool emoji].” as well as skepticism toward the perceived normalization of the virus: “Are we supposed to believe that all the spectators at the World Cup venue are cheering through 40-degree fevers?”

These humorous expressions are often categorized as positive by sentiment lexicons due to their playful vocabulary. However, inspection of sentiment distributions in these threads revealed that neutral sentiment was predominant, suggesting that while the humor mechanism mitigates distress, it is not sufficient to transform the overall public sentiment toward a positive state.

### Risk stories: from affect amplifier to solidarity catalyst

Risk stories were originally thought to elicit more negative emotions, but this study surprisingly found that risk messages containing risk stories induced fewer negative emotions.

The period in late 2022 represented a unique phase of the COVID-19 pandemic in China, marked by a rapid policy transition and a peak in infections. However, the crisis sparked a sense of solidarity among people. According to Durkheim’s theory of collective effervescence (Durkheim, 1912), when members of a society synchronize their thoughts and actions during upheavals, they revive their sense of social belonging and shared beliefs, generating positive emotional energy. Garcia and Rimé (2019) provided large-scale empirical evidence that this process operates equally in digital spaces, demonstrating that collective emotional sharing following a terrorist attack led to long-term increases in solidarity indicators and positive affect among participants.

A review of Zhihu discussions revealed that when users shared their infection experiences and emotions, others responded with prevention tips, treatment advice, and emotional support, shifting the focus from threat perception to mutual reassurance. For instance, comments like “I had a fever last night, drank soybean water, and felt better this morning—give it a try!” or “Brother, are you still there? Let us know you are safe.” reflect a supportive and helpful mindset that mitigated the negativization of collective emotions.

This dynamic may be further amplified in collectivist societies such as China, where individuals tend to perceive themselves as integral components of a social relational network (Hofstede, 1980, 2011), potentially making the solidarity-generating potential of shared risk narratives even more salient. Risk stories thus functioned as catalysts for mutual support rather than amplifiers of negative affect. This identifies an important sociocultural boundary condition for the Visschers et al. (2012) framework, demonstrating that the affect-inducing potential of risk stories is contingent on the broader cultural and situational context in which risk communication occurs.

### Outrage factors and visual elements: diluted potency and constrained vividness

The absence of significant effects for both outrage factors and visual elements suggests that the mere presence or accumulation of these cues does not straightforwardly translate into greater prevalence of negative emotion.

The non-significant effect of outrage factors warrants closer examination. Sandman (1993) originally identified 20 outrage factors as drivers of public emotional responses to risk, but the framework does not assume that each factor carries equal emotional weight. In the present study, outrage factors were operationalized as an additive count of 12 items, an approach that has precedent in prior research (Ju et al., 2015; Ju, 2024) but implicitly assumes equal emotional potency across all factors.

Ju (2024), however, provides direct evidence challenging this assumption. While the aggregate outrage score significantly predicted emotional expressions among news readers, the effects of individual factors were highly uneven, with only a small subset driving the overall association. This suggests that the emotional mobilizing capacity of outrage factors is not uniformly distributed, and that simple aggregation may obscure meaningful variation, effectively canceling out the influence of more potent individual factors when they are diluted by the presence of less emotionally charged ones. Which specific factors carry greater weight may also vary across risk contexts and communication environments, introducing a degree of contingency that additive scoring is ill-equipped to capture. Future research would benefit from moving beyond aggregate measurement toward approaches that are sensitive to the differential emotional valence of individual outrage factors.

Regarding visual elements, the theoretical basis for H4 rests on the Risk as Feelings hypothesis, which holds that the emotional impact of visual content derives primarily from its capacity to generate vivid mental imagery of threatening consequences (Loewenstein et al., 2001; Holmes and Mathews, 2005). The non-significant finding suggests that this mechanism was largely inoperative in the present context. As a knowledge-sharing platform catering predominantly to highly educated young users, the visual content published on Zhihu regarding the XBB variant consisted largely of informational charts, research illustrations, and data visualizations with very few emotionally evocative depictions of suffering or threat. Consistent with the coding scheme adopted in this study, such objective visual material was excluded from the measure of affect-inducing visual elements. The descriptive statistics reflect this platform reality, with visual elements recording a mean of only 0.26 across the sample. With vividness-inducing imagery largely absent from the dataset, the affective pathway through which visual elements are theorized to operate had little opportunity to manifest.

Building on these findings, this study offers both theoretical and practical implications for digital health communication research and practice.

This study makes three theoretical contributions. First, by introducing affect-inducing cues as structural predictors of collective negative emotion prevalence on social media, this study constructs an explanatory framework that connects risk message properties to collective emotional outcomes during public health emergencies. This represents a substantive advance toward understanding not merely the state and trajectory of collective emotions, but the message-level mechanisms that drive them.

Second, this study provides direct empirical tests of the Visschers et al. (2012) affect-inducing cues framework with emotional responses as the outcome variable, responding to their explicit call for research that moves beyond risk perception measures. The selective pattern of results, with fear appeals and risk stories emerging as significant predictors while outrage factors and visual elements did not, offers a more granular empirical understanding of which cues reliably shape collective emotional responses, advancing the framework beyond its largely review-based origins.

Third, by extending the framework into social media UGC environments and a collectivist cultural context, this study identifies layered contextual boundary conditions on its predictions. The emotional function of risk stories was fundamentally transformed under the conditions of China’s late-2022 pandemic transition, while the non-significant effects of outrage factors and visual elements further reflect the differential potency of individual outrage cues and the platform affordances of Zhihu, respectively. These findings collectively demonstrate that the affect-inducing potential of risk communication cues is highly context-dependent, and that the framework requires careful contextual calibration when applied beyond its original one-way communication setting.

Beyond these theoretical contributions, the findings also carry practical implications for public health authorities. The inverted U-shaped relationship between fear appeal intensity and negative emotion prevalence suggests that an optimal intensity range exists for fear-based risk messaging. Within this range, fear appeals effectively elevate collective negative emotions, maintaining public alertness to the risk. Beyond this threshold, however, the emotional dynamic shifts: rather than sustaining negative affect, high-intensity fear appeals may activate defensive processes that ultimately suppress emotional arousal, reducing rather than reinforcing public vigilance toward the risk. Communicators should therefore calibrate fear appeal intensity with care, pairing threat information with efficacy-enhancing content to support adaptive coping. The emotion-attenuating effect of risk stories, meanwhile, highlights their underutilized potential as tools for emotional management during crises. In collectivist societies, facilitating the sharing of personal experiences and mutual support may sustain social cohesion more effectively than suppressing such narratives. Finally, the limited emotional impact of outrage factors and visual elements suggests that regulatory resources are better directed toward monitoring fear appeal intensity than toward blanket restrictions on emotionally evocative content.

### Limitation and future study

Several limitations of this study should be acknowledged, which also provide directions for future inquiry. First, methodologically, the sentiment analysis employed in this research relies on established lexicons and semantic rules for a three-way classification of emotional valence. While effective for determining general sentiment in large-scale short texts, this approach has inherent constraints in capturing more complex emotional nuances such as ambivalence, and may also overlook sophisticated linguistic features like deep irony or sarcasm. Future research should consider adopting multi-label sentiment classification frameworks that allow a single comment to carry multiple emotional labels simultaneously.

Second, the distribution of the sample data and its specific temporal context present certain constraints. The intensity of fear appeals within the collected threads was primarily concentrated in the low to moderate range, with a relatively small number of threads exhibiting very high fear appeal intensity. This limited representation at the far end of the spectrum may affect the statistical power to fully map the inverted U-shaped curve. Furthermore, the findings are situated within a unique societal backdrop characterized by a historic transition in pandemic policy and a subsequent nationwide peak in infections. This heightened baseline of collective anxiety and the shared experience of illness likely moderated the impact of risk cues, particularly the observed solidarity in response to risk stories. Additionally, the exclusive focus on Zhihu limits the generalizability of the findings. Its predominantly highly educated, middle-class user base may not be representative of broader public emotional reactions during health crises. The platform lacked the highly emotional or vivid photographs typically found in other risk communication contexts, which constrained the examination of visual elements and limits generalizability across diverse digital environments. Future studies should adopt cross-platform comparisons to validate these patterns.

Finally, while this study identifies how external message properties drive collective emotions, it pays less attention to the internal dynamics of emotional interaction. Collective emotions are not only shaped by initial risk cues but also by horizontal processes such as emotional contagion and group polarization (Parkinson, 2011; Del Vicario et al., 2016; Goldenberg et al., 2014). Although this model controlled for discussion duration, it does not fully account for how these internal social influences interact with message level factors over time. To develop a more robust explanatory framework, future research should integrate these interpersonal dynamics with structural message properties to better understand the complete formation and evolution of collective affect during public health emergencies.

## Data Availability

The raw data supporting the conclusions of this article will be made available by the authors, without undue reservation.
