# Supplementary material for: Affect-inducing cues in risk messages: impact on collective negative emotions during public health emergencies
Source: Front Psychol. 2026 May 28;17:1829692. doi: 10.3389/fpsyg.2026.1829692 (PMC13254828; doi:10.3389/fpsyg.2026.1829692)
Supplement: Supplementary file 1 [file Data_Sheet_1.pdf]

Affect-Inducing Cues in Risk Messages: Impact on Collective Negative Emotions During Public Health Emergencies

Supplemental materials

S1 - Sentiment analysis

Several well-established Chinese sentiment dictionaries exist, including the HowNet Sentiment Dictionary from CNKI.net, the National Taiwan University Semantic Dictionary, and the sentiment vocabulary ontology database developed by the Dalian University of Technology. However, the entries in these dictionaries are generally broad or commonly used, lacking specificity to the pandemic context. Therefore, this study seeks to mine affective words closely related to COVID-19 and expand the dictionary to enable more accurate lexicon-based sentiment analysis of Zhihu comments.

Affective Words Mining and Lexicon Expansion

The basic sentiment lexicon used in this study is the sentiment vocabulary ontology database developed by the Dalian University of Technology (Xu et al., 2008). This lexicon classifies words into three polarities—neutral, positive, and negative—and into seven discrete emotion categories: joy (乐), goodness/approval (好), anger (怒), sadness (哀), fear (惧), disgust (恶), and surprise (惊). Each word is also assigned an intensity level of 1, 3, 5, 7, or 9, with 9 representing the highest and 1 the lowest, where intensity reflects the strength of the assigned discrete emotion.

To identify new affective words, Wang et al. (2015) proposed three indicators: word frequency, internal coupling, and the information entropy of neighboring characters.

Word Frequency

For a word to be included in the lexicon, it must first exhibit a high frequency of occurrence in the texts. In this study, the median frequency of known affective words from

the sentiment lexicon, as they appeared in the Zhihu comments, was used as the threshold. Any word with a frequency exceeding this threshold was considered a potential new affective word.

Inside Coupling

Inside coupling (IC) reflects the degree of internal cohesion within a word (Wang et al., 2015). In Chinese, a word typically consists of at least two characters. When such a word is split into two parts, there are often multiple possible segmentations. For example, the word “好开心” (“so happy”) can be divided as {("好开", "心"), ("好", "开心")}. Let all possible dichotomies of a word w be represented as $\{(w_{11}, w_{12}), (w_{21}, w_{22}), (w_{31}, w_{32})\ldots\ldots(w_{n1}, w_{n2})\}$. The formula for calculating the inside coupling of w is as follows:

$$\text{inside coupling (w)} = \frac{1}{n}\sum_{i=1}^{n}\frac{p(w)}{p(w_{i1})\times p(w_{i2})} \quad (1)$$

(Adapted from Wang et al., 2015)

p(w) denotes the probability of occurrence of w in the entire corpus, which can be calculated as:

$$p(w) = \frac{N(w)}{N_D} \quad (2)$$

(Adapted from Wang et al., 2015)

N(w) denotes the frequency of word w, and N_D refers to the total word count of all comments. For a given word, a higher inside coupling value indicates a tighter connection between its constituent characters. In this study, the median inside coupling of known affective words

was used as the threshold; any word with an IC value exceeding this threshold was considered to meet one of the criteria for being identified as a new affective word.

Information Entropy of the Neighbouring Characters

The ways in which a word is used in texts should be diverse, meaning that "the characters adjacent to the word would be highly uncertain" (Wang et al., 2015, p. 220). Information entropy can be applied to quantify this uncertainty. For a given word w, let the set of all characters that ever appear immediately to its left (or right) in the texts be $\{c_1, c_2, c_3 \ldots\ldots c_n\}$. The information entropy of the neighbouring characters to the left (or right) of w is then calculated as:

$$\mathrm{IE(w)} = -\sum_{i=1}^{n} \frac{n_i}{n} \log_2 \frac{n}{n_i} \quad (3)$$

(Adapted from Wang et al., 2015)

Here, n_i denotes the number of times c_i appears as the left (or right) neighbouring character of w, and n represents the total number of occurrences of all characters in the neighbouring set C as the left (or right) neighbour of w (Wang et al., 2015). The smaller of IEleft(w) and IEright(w) will be taken as the final indicator value. And the median information entropy of those known affective words will be set as the threshold likewise.

Based on the three indicators described above and the predetermined thresholds, a set of new affective words was identified. The next step was to apply point-wise mutual information (PMI) to calculate the sentiment scores of these new words (Wang et al., 2015; Kaji & Kitsuregawa, 2007). The underlying principle is that the higher the probability of two words appearing in the same document, the stronger their association. The formula is:

$$\mathrm{PMI}(w_1, w_2) = \log_2 \frac{p(w_1, w_2)}{p(w_1) \times p(w_2)} \quad (4)$$

(Adapted from Wang et al., 2015)

$P(w_1, w_2)$ refers to the probability of two words appearing in the same comment, while $p(w_1)$ and $p(w_2)$ denote the probabilities of each word occurring individually (Wang et al., 2015).

In the present study, the 35 most frequent positive and 35 most frequent negative sentiment words in the collected Zhihu comments were selected to form the positive word set (W_P) and the negative word set (W_N), respectively. For each newly identified affective word, its sentiment score was obtained by calculating the sum of its PMI values with all words in W_P and subtracting the sum of its PMI values with all words in W_N.

If its score is positive, the word is classified as a positive word; if the score is negative, the word is classified as negative; and if the score equals 0, the word is considered neutral. Based on these scores, the new words are then assigned to one of three polarities, matching the structure of the basic lexicon described earlier.

Similarly, discrete emotion categories were determined using the same approach, with PMI calculations performed separately for the representative word sets corresponding to each emotion category. The emotion category with the highest PMI value was assigned as the word's primary category, and its intensity level was determined by mapping the absolute value of the PMI sum for that category to the five intensity levels of the basic lexicon (1, 3, 5, 7, 9).

In this way, the sentiment lexicon is expanded and made more suitable for analyzing pandemic-themed texts.

Sentiment Lexicon Construction Results

Using the sentiment lexicon expansion method, new emotional words were extracted from 12,894 comments. Thresholds for word frequency (1), inside coupling (427.2), and neighborhood entropy (0.0) were determined based on the median values of 1,613 known emotional words. These parameters were calculated using Python. Applying the thresholds, 4,864 new affective words were identified in addition to those contained in the Dalian University of Technology's sentiment ontology library.

PMI analysis classified these words into 1,359 negative, 2,420 neutral, and 1,085 positive entries, with 586 categorized under "fear." As shown in Table 1, terms such as "variant," "rural," "incubation period," "mask," "hospital," and "asymptomatic"—ordinarily neutral in everyday contexts—acquired emotional connotations in discussions about "Xbb."

Table 1

Examples of new emotional words

Word	Polarity	Intensity	Emotion Type	Word	Polarity	Intensity	Emotion Type
酒精 (alcohol)	Positive	7	Disgust	诺氟沙星 (norfloxacin)	Negative	0	Surprise
治疗 (treatment)	Positive	5	Good	防毒面具 (gas mask)	Negative	0	Sadness
转阴	Positive	5	Good	纸尿裤	Negative	-1	Disgust

(test negative)				(diaper)			
咳嗽药 (cough medicine)	Positive	5	Happiness	症状 (symptom)	Negative	-3	Disgust
口罩 (mask)	Positive	3	Good	腹泻 (diarrhea)	Negative	-3	Disgust
抵抗力 (immunity)	Positive	3	Happiness	潜伏期 (incubation)	Negative	-3	Fear
维生素 (vitamin)	Positive	1	Fear	传播 (spread)	Negative	-5	Fear
怀孕 (pregnancy)	Positive	0	Disgust	变异 (variant)	Negative	-5	Fear
冰袋 (ice pack)	Positive	0	Disgust	农村 (rural)	Negative	-7	Sadness
蛋白粉 (protein powder)	Positive	0	Good	心脏 (heart)	Negative	-9	Disgust

Identifying Grammatical Relations Between Words

The polarity value (positive = 1, negative = −1, neutral = 0)) of each word is obtained from the optimized lexicon. Stanford Parse (https://nlp.stanford.edu/software/lex-parser.shtml) is then used to identify the grammatical relations—referred to as dependencies—between words within a simple sentence (Wang et al., 2015; Wu & Lu, 2019) (see Table 2).

Table 2
Samples of dependencies parsed by Stanford NLP

Name	Relationship	Sentence	Dependencies
nsubj	object and subject	我爱(I love)	(love, I)
dobj	object and predicate	吃面包(eat bread)	(eat, bread)
amod	adjectival modifier	面包好吃(the bread is yummy)	(bread, yummy)
advmod	adverbial modifier	非常快(very quickly)	(quickly, very)
neg	negation modifier	不高兴(not happy)	(happy, not)

Since degree adverbs can indicate the extent to which an adjective or verb applies, they can amplify or diminish a word's polarity value, while negations can reverse its polarity. Therefore, when a word is modified by a degree adverb or a negation, its value is multiplied by the corresponding weight of that adverb or negation.

The HowNet lexicon includes a degree adverb dictionary with six levels, each assigned a specific weight (Wu & Lu, 2019) (see Table 3). For negations, if there is only one negation relation, the sentiment polarity is reversed (value multiplied by –1); if there are two negations (double negation), the polarity remains unchanged (multiplied by 1). The initial polarity value

of a clause is calculated as the sum of the adjusted values of all words within the clause (Wang et al., 2015; Wu & Lu, 2019).

Table 3

Samples of degree adverbs and weights

Level	Word Sample	Weight	Total
over	过度(excessively)，超(super)	3	30
extreme	非常(highly)，极度(extremely)	2.5	69
very	大为(greatly)、格外(especially)	2	42
more	进一步(further)、较为(comparatively)	1.5	37
-ish	些微(slightly)、或多或少(more or less)	1	29
insufficiently	不怎么(not really)	0.5	12

Applying Semantic Rule Sets

Different clause relations give different weights to clauses in a complex sentence, and this is called inter-clause rules. The identification of clause relations is based on conjunctions that signal these relations Summarizing Wang et al. (2015), Table 4 presents the assigned weights between clauses triggered by conjunctions that indicate different types of inter-clause relations. Each clause's polarity value is weighted and summed to produce the initial polarity value of the complex sentence.

Finally, sentence-type rules are applied to assign weights to different sentence types—primarily interrogative, rhetorical, declarative, and exclamatory. Sentence type is

identified according to the punctuation mark at the end of each complex sentence (Wu & Lu, 2019). The final polarity value of a text is obtained by summing the values of all sentences. A positive value indicates positive sentiment, whereas a negative value indicates negative sentiment.

Table 4

Clause weighting rules based on inter-clause relations

Relationship	Condition	Weight Assignment	Conjunction Examples
Adversative - Preceding Connector	Only a preceding conjunction appears	Clauses before connector: weight = 1; clauses after connector: weight = 0	虽然、即便、尽管……(although)
Adversative - Following Connector	Only a following conjunction appears	Clauses before connector: weight = 0; clauses after connector: weight = 1	但、但是、可、可是……(but)
Adversative - Paired Connectors	Paired adversative conjunctions appear	Clauses before connector: weight = 0; clauses after connector: weight = 1	-
Progressive	Progressive conjunction appears	First clause: Weight = 1; following clause: weight = 1.5; and each subsequent clause increases its weight by 0.5	而且、更重要的是、更别说…… (and, more importantly, not

		relative to the previous clause	to mention)
Conditional	Conditional conjunction appears	Clauses before connector: weight = 1; clauses after connector: weight = 0.5	那么、那、则……(then, thus, in that case)
Negative Conditional	Negative conditional conjunction appears	Clauses before connector: weight = -1; clauses after connector: weight = -0.5	如果不、如果没有、倘若并非……(if not)

S2 - Criteria for Selecting Outrage Factors

Covello and Sandman (2004) initially identified 20 outrage factors and provided their definitions. However, some of these factors overlap conceptually. For example, “understanding” and “uncertainty” are similar to “familiarity,” as all assess whether a risk can be easily explained and is supported by sufficient information; “trust” overlaps with “ethical/moral nature” in evaluating whether a risk arises from legitimate motives aligned with public values; and “effects on future generations” is similar to “effects on children” (Covello & Sandman, 2004; Li & Zhong, 2022; Ju et al., 2015). These four factors were therefore removed.

In addition, several factors were deemed less applicable to the COVID-19 “Xbb” risk. For instance, “benefits,” “ethical/moral nature,” and “human vs. natural origin” all relate to the origin of the risk, that is, whether it stems from economic gain, unethical behavior, or human causes (Covello & Sandman, 2004). By 2023, in the later stage of the pandemic, the question of COVID-19’s origin had lost public attention in China, where the focus had shifted to the impact of new variants on life safety during high infection periods. These three factors were thus excluded. Finally, the “media attention” factor, which assesses whether a risk event receives media coverage, was also excluded, as this study specifically examines user discussions of the risk on social media.

As a result, 12 outrage factors were retained for analysis.

References:

Covello, V. T., and Sandman, P. M. (2001). “Risk communication: evolution and revolution,” in *Solutions to an Environment in peril*, ed. A. Wolbarst (Baltimore, MD: The Johns Hopkins University Press), 164 – 178.

Ju, Y., Lim, J., Shim, M., & You, M. (2015). Outrage factors in government press releases of food risk and their influence on news media coverage. *Journal of health communication*, 20(8), 879-887.

Kaji, N., and Kitsuregawa, M. (2007). Building lexicon for sentiment analysis from massive collection of HTML documents. In *Proceedings of the 2007 Joint Conference on Empirical Methods in Natural Language Processing and Computational Natural Language Learning (EMNLP-CoNLL)* (Prague, Czech Republic: Association for Computational Linguistics), 1075 – 1083.

Li, S. S., and Zhong, Y. (2022). Tufa gonggong weisheng shijian zhong wuhan qingnian fengxian ganzhi de "fennu xiaoying"——jiyu shejiao meiti shiyong de jiaohu zuoyong de kaocha [Outrage Effects on the Risk Perception of COVID-19 Moderated by Social Media Use of Wuhan Youth]. *Xinwen yu chuanbo pinglun*, 75(04), 74 – 86. doi: 10.14086/j.cnki.xwycbpl.2022.04.006

Wang, Z. T., Yu, Z. W., and Lu, X. J. (2015). Jiyu cidian he guizeji de zhongwen weibo qinggan fenxi [Sentiment analysis of Chinese micro blog based on lexicon and rule set]. *Jisuanji gongcheng yu yingyong*, 51(08), 218 – 225. doi:

10.3778/j.issn.1002-8331.1308-0187

Wu, J. S., and Lu, K. (2019). Jiyu duobu qinggan cidian he guizeji de zhongwen weibo qinggan fenxi yanjiu [Chinese Weibo Sentiment Analysis Based on Multiple Sentiment Lexicons and Rule Sets]. *Jisuanji yingyong yu ruanjian*, 36(09), 93 – 99. doi: 10.3969/j.issn.1000-386x.2019.09.017

Xu, L. H., Lin, H. F., Pan, Y., Ren, H., & Chen, J. M. (2008). Qinggan cihui benti de gouzao[Constructing the affective lexicon ontology]. *Qingbao xuebao*, 27(2), 180-185
